# The Th17/IL-17 axis and host defence against fungal infections

**DOI:** 10.1016/j.jaip.2023.04.015

**Published:** 2023-04-26

**Authors:** Stuart G Tangye, Anne Puel

**Affiliations:** 1 Garvan Institute of Medical Research, Darlinghurst, NSW, Australia; 2 School of Clinical Medicine, UNSW Faculty of Medicine & Health, Darlinghurst 2010, NSW, Australia; 3 Laboratory of Human Genetics of Infectious Diseases, Necker Branch, INSERM U1163, Necker Hospital for Sick Children, Paris, France.; 4 University of Paris, Imagine Institute, Paris, France.; 5 St. Giles Laboratory of Human Genetics of Infectious Diseases, Rockefeller Branch, The Rockefeller University, New York, NY, USA.

**Keywords:** Th17 cells, IL-17 cytokines, chronic mucocutaneous candidiasis, inborn errors of immunity, anti-fungal immunity, anti-cytokine autoantibodies, STATs

## Abstract

Chronic mucocutaneous candidiasis (CMC) was recognized as a primary immunodeficiency in the early 1970’s. However, for almost 40 years, its genetic etiology remained unknown. The progressive molecular and cellular description of inborn errors of immunity (IEI) with syndromic CMC pointed toward a possible role of IL-17-mediated immunity in protecting against fungal infection and CMC. Since 2011, novel IEI affecting either the response to or production of IL-17A and IL-17F (IL-17A/F) in patients with isolated or syndromic CMC provided formal proof of the pivotal role of the IL-17 axis in mucocutaneous immunity to *Candida* spp. And, to a lesser extent, to *Staphylococcus aureus* in humans. In contrast, IL-17-mediated immunity seems largely redundant against other common microbes in humans. In this review, we outline the current knowledge of IEI associated with impaired IL-17A/F-mediated immunity, highlighting our current understanding of the role of IL-17A/F in human immunity.

## INTRODUCTION

Cytokines are proteins produced by many cell types and have critical roles in immunity. Specifically, cytokines regulate the development, activation, and differentiation of leukocytes and non-haemopoietic cells. These processes underlie protection against infectious diseases following natural pathogen infection or immunization, thereby providing the host with long-lived immunological memory. In contrast, aberrant cytokine signaling can cause immune dysregulation, eg early-onset autoimmunity. Thus, balanced signals provided by distinct cytokines, and delivered to specific cell subsets, are critical for immune homeostasis^1–4^.

Approximately 60 different cytokines have been identified, including interleukins (IL-1 through IL-40), interferons, transforming growth factors and members of the TNF superfamily^1^. Most cytokines have pleiotropic effects on different immune cells; there is also substantial overlap in their function. The biological effects of many cytokines are mediated by JAK/STAT signalling pathways^2, 5–7^. Four JAKs (JAK1, JAK2, JAK3, Tyk2) and seven STATs (STAT1, 2, 3, 4, 5a, 5b, 6) have been identified in mammalian genomes^2, 5, 6^. JAKs associate with the cytoplasmic domains of cytokine receptors and, following engagement by specific ligands, phosphorylate tyrosine residues to provide docking sites for STATs. Receptor-associated STATs undergo JAK-mediated phosphorylation, resulting in formation of multimers that translocate to the nucleus and bind specific DNA sequences, thereby regulating expression of target genes^2, 5–7^.

The essential and non-redundant functions of specific cytokines in host defense and immune dysregulation in humans have been elegantly and repeatedly revealed by the discovery of individuals with pathogenic variants in genes encoding cytokines, cytokines receptors, or downstream transcription factors that manifest as immunodeficient and/or autoimmune states^4, 8, 9^. Currently, variants, either loss-of function (LOF) or gain-of function (GOF), have been identified in genes encoding cytokines (*IL10*, *IL17F*, *IL12B*, *IL21*), cytokine receptors (*IL2RA*, *IL2RB*, *IL2RG*, *IL7RA*, *IL6R*, *IL6ST*, *IL10RA*, *IL10RB, IL11RA*, *IL12RB1*, *IL12RB2*, *IL17RA*, *IL7RC*, *IL21R*, *IL23R, IFNGR1*, *IFNGR2, IFNAR1, IFNAR2)*, JAKs (*JAK1*, *JAK3*, *TYK2*), transcription factors activated by specific cytokines (*STAT1*, *STAT2*, *STAT3*, *STAT5B*, *STAT6*), and transcription factors that regulate lymphocyte fate (*FOXP3*, *RORC*, *ZNF341*, *IRF1*, *IRF4*), resulting in aberrant development and/or effector function of different human immune cells^4, 8–11^. These discoveries have not only defined genetic causes of unique inborn errors of immunity (IEI) but also established key roles for cytokine signalling and cell types in host defense against specific pathogens. Remarkably, infectious complications that define some IEI can also result from production of neutralising anti-cytokine autoantibodies which impede the function of specific cytokines^12^. Here, we provide an overview of how the study of IEI has contributed to our understanding of the molecular and cellular requirements for the generation and function of human IL-17-producing cells in health and disease, and how these findings reveal mechanisms of disease pathogenesis that may enable improved management and treatment of individuals with these conditions.

## IL-17A and IL-17F production, regulation, and signaling.

Interleukin (IL)-17A, also named IL-17, belongs to the IL-17 family of cytokines, which includes IL-17B, IL-17C, IL-17D, IL-17E (also called IL-25) and IL-17F^13^. The founding and best characterized member - IL-17A - was cloned in 1993 and initially named cytotoxic T lymphocyte-associated antigen 8 (CTLA-8)^14, 15^. IL-17F is the most closely related member, sharing 55% similarity with IL-17A. Both IL-17A and IL-17F are usually produced by the same cell types^16^, and function as homodimers (IL-17A/IL-17A, IL-17F/IL-17F) or heterodimers (IL-17A/IL-17F)^13, 17–19^. However, IL-17A homodimers more potently induce production of pro-inflammatory cytokines and chemokines than IL-17F homodimers by target cells expressing IL-17 receptors, such as fibroblasts, macrophages, or epithelial cells. IL-17A/IL-17F heterodimers have intermediate signaling strength^17–19^.

## CD4^+^ T cell differentiation

CD4^+^ T cells play critical roles in mediating immunity against a broad array of pathogens that cause serious infectious diseases. This reflects the extraordinary ability of CD4^+^ T cells to differentiate into distinct effector subsets that co-operate with other immune cells to protect the host against infection with specific pathogens. Naïve CD4^+^ T cells can differentiate into distinct subsets of cells that have unique roles in protection against infectious diseases^3, 20^. For instance, distinct and specialised CD4^+^ T cell subsets have been implicated in immunity against viral or intramacrophagocytic (Th1), parasitic (Th2) and fungal (Th17) infections, as well as regulating B-cell differentiation/humoral immunity (Tfh) and immune regulation/homeostasis (Tregs)^3, 20^. CD4^+^ T cell differentiation is mediated by the microenvironment in which CD4^+^ T cells encounter various signals provided by antigen-presenting cells (APCs). These take the form of MHC class II/peptide complexes and co-stimulatory signals provided by cell-cell interactions and cytokines which activate relevant signalling pathways and induce lineage-specific transcription factors^3, 20^. These pathways not only induce commitment of naïve CD4^+^ T cells to a specific fate, but also restrain differentiation of naïve CD4^+^ T cells to alternate effector fates. Thus, the molecular wiring of CD4^+^ T cells allows these cells to acquire and retain specialised functions to establish and maintain effective protection against pathogens^3, 20^.

## Th17 and IL-17-producing cells

In 2005, a CD4^+^ T helper (Th) subset distinct from Th1 and Th2 cells was described in mice as the main source of IL-17A and IL-17F and was therefore designated Th17^21–24^. Since then, IL-17A and IL-17F have been found to be produced by additional immune cells in humans and mice, including cytotoxic CD8^+^ T (Tc)17 cells^25–27^, γδ T cells^28–30^, type 3 innate lymphoid cells (ILC)3^31–35^, invariant natural killer T (iNKT)^36–38^, mucosal-associated invariant T (MAIT) cells^39–41^, and Th1* cells^42^. Here, we will predominantly focus on IL-17-producing CD4^+^ Th17 cells.

**Figure 1:**
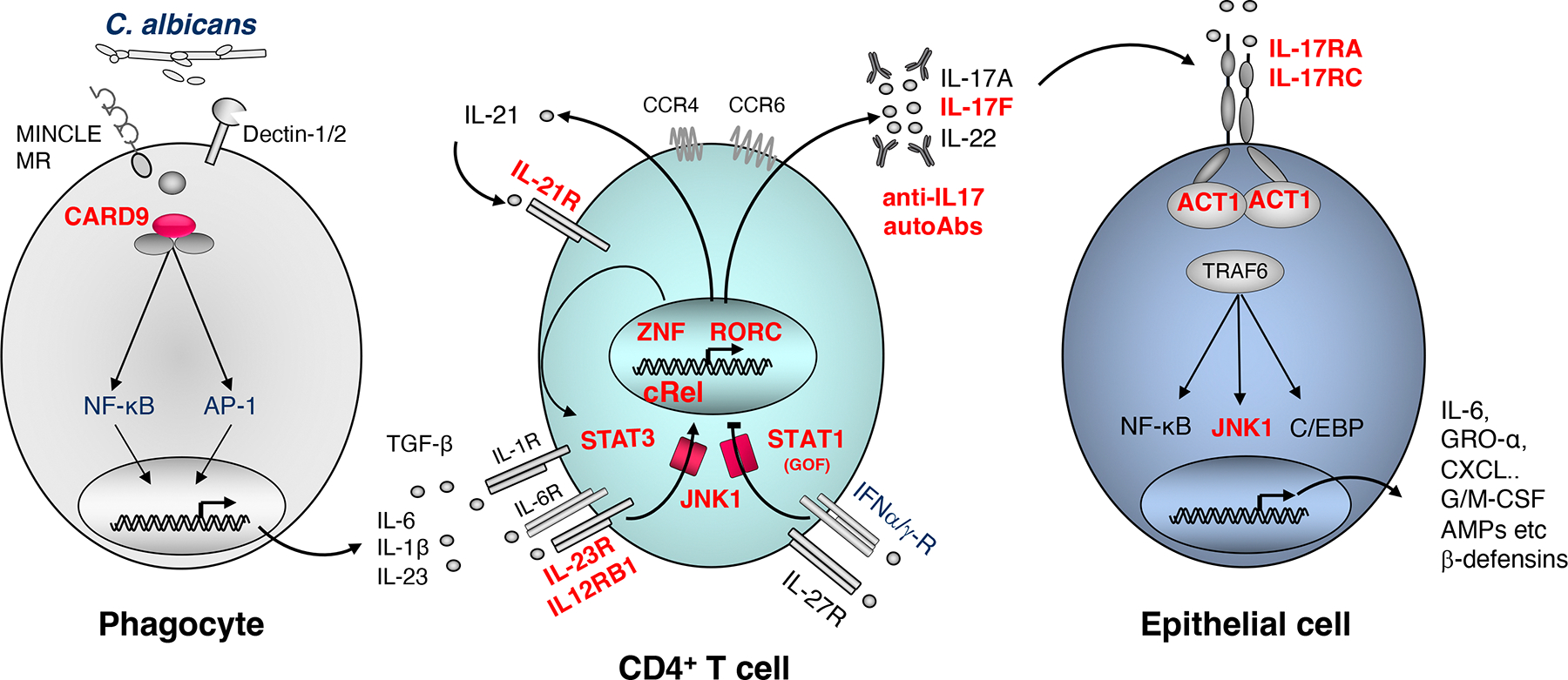
Inborn errors of immunity and phenocopies that disrupt IL-17-mediated immunity against fungal infections. In response to cytokines produced by myeloid cells, naïve CD4^+^ T cells differentiate into specialised subsets of effectors cells. Production of canonical cytokines enables elicitation of specific effector functions important for host defence against pathogen infections. In the setting of Th17 cells, integration of STAT3/ZNF341-dependent signals downstream of cytokines including IL-23 and IL-21 in CD4+ T cells leads to expression of the transcription factor RORγt and induction of the Th17 program. IL-17 cytokines activate epithelial cells via IL-17R/ACT1/JNK1, resulting in production of pro-inflammatory and anti-microbial peptides. Molecules shown in red have been found to be mutated in specific IEI and impair either the generation or function of Th17 cells causing CMC (and often *Staph* infection). Autoantibodies against IL-17A/IL-17F phenocopy inborn errors of IL-17-mediated immunity.

Th17 cells have been well-characterised based on expression of surface receptors (CCR4, CCR6, CD161, IL-23R) and Th17-lineage specific transcription factors RORγt and RORα, which regulate production of canonical cytokines including IL-17A, IL-17F, IL-21 and IL-22 (and IL-26 in humans) and CCL20^3, 20^ (Figure 1). Due to the production of pro-inflammatory cytokines, Th17 cells have been implicated in numerous autoimmune conditions, such as inflammatory bowel disease, psoriasis, and ankylosing spondylitis^43^. However, before IL-17 was identified as a canonical Th17 cytokine, studies in mice demonstrated a key role for IL-17A/IL-17AR signaling in host immunity against fungal infections^44^. This was extended to reveal that Th17 cells were a predominant source of the IL-17 required for mediating anti-fungal immunity in mice^45^. Seminal studies in humans also found that memory CD4^+^ T cells co-expressing CCR6 and CCR4 were enriched for (1) production of IL-17 and expression of *RORC* (encoding the transcription factor RORγt), and (2) *Candida*-specific CD4^+^ T cells^46^. These studies established a fundamental role for IL-17-producing Th17 cells in host defense against mucosal fungal infections in mice and humans. Mechanistically, IL-17 induced anti-fungal immunity by binding IL-17 receptors expressed on epithelial cells and inducing production of anti-microbial peptides and β-defensins by these cells^47^ (Figure 1). Consistent with these observations, individuals with IEI that compromise IL17-mediated immunity develop chronic mucocutaneous candidiasis (CMC). Analysis of these conditions have provided invaluable insights into the pathways that regulate Th17 cell differentiation and function^48^.

## Molecular requirements for generating human Th17 cells

### Transcription Factors:

Naïve CD4^+^ T cells give rise to specialised subsets following receipt of instructive signals provided by the cellular microenvironment established during T cell activation. Numerous studies, performed predominantly in mice, identified various cytokines and transcription factors required for in vivo and in vitro Th17 cell differentiation and maintenance, including the cytokines TGF-β, IL-1, IL-6, IL-21, IL-23^23, 42, 49–57^, and transcription factors STAT3^58–60^, RORγt^61^, and RORα^62^. However, several of these findings have not been confirmed by other investigators^63, 64^. IL-6, IL-21, and IL-23 activate STAT3, providing the basis for the initial discovery in mice that STAT3 plays a critical role in generating murine Th17 cells^58–60^. Autosomal dominant hyper IgE syndrome (AD-HIES) is caused by heterozygous germline dominant negative *STAT3* (STAT3^DN^) variants and is characterised by recurrent opportunistic bacterial and fungal infections^65–67^. Indeed, >85% of affected individuals suffer from CMC, and >90% from pneumonia caused by *Staphylococcus aureus* or *Streptococcus pneumoniae*^67^ (Table 1). Analysis of STAT3-deficient patients revealed a lack of Th17 cells *ex vivo*^68–72^, as determined by significant decreases in CCR6^+^ CD4^+^ T cells, and impaired production of IL-17A, IL-17F, and IL-22 by memory CD4^+^ T cells under neutral and Th17-inducing culture conditions^71^. This defect was cell intrinsic, because naïve STAT3^DN^ CD4^+^ T cells were unable to upregulate expression of *RORC* and differentiate into IL17A/IL-17F-secreting cells *in vitro* following culture under Th17-polarising conditions^68, 73^ (Table 1, Figure 1). Interestingly, STAT3^DN^ patients had numerical reductions in MAIT cells, and the residual MAIT cells present were impaired in production of Th17 cytokines^41^. As these innate-type T cells recognise fungal antigens, it is possible that a deficiency in these cells also contributes to compromised anti-fungal immunity in AD HIES.

Individuals have also been identified with recessive variants in *RORC*^74^. Perhaps not surprisingly, most (>80%) patients with RORγt deficiency develop CMC (Table 1). LOF *RORC* mutations abolished the generation of Th17 and MAIT cells *in vivo* and *in vitro*^74^ (Table 1), consistent with findings from STAT3^DN^ patients whose CD4^+^ T cells failed to upregulate *RORC* to generate Th17 cells^68, 73^, and lacked MAIT cells^41^. Furthermore, while *Candida* specific CD4^+^ T cells could be generated in the absence of RORγt, these cells were completely devoid of production of Th17 cytokines^74^ (Figure 1). Interestingly, proportions of CCR6^+^ CD4^+^ memory T cells were intact in RORγt-deficient patients^74^, indicating that STAT3 signalling regulates CCR6 expression independently of RORγt.

An autosomal recessive (AR) form of HIES that clinically phenocopies STAT3^DN^ HIES, including high penetrance of CMC, is caused by bi-allelic mutations in the transcription factor *ZNF341*^75, 76^ (Table 1). The mechanistic link between ZNF341 and STAT3 function was provided by the finding that ZNF341 binds the *STAT3* promoter and is essential for *STAT3* transcription-dependent autoinduction and sustained STAT3 activity. Consequently, ZNF341-deficient patients have low levels of *STAT3* mRNA and protein and poor responses following stimulation with STAT3-activating cytokines^75, 76^. ZNF341-deficient patients had a paucity of CD4^+^ CCR6^+^ memory T cells. The lack of Th17-phenotype cells was confirmed functionally by demonstrating reductions in proportions of memory CD4^+^ T cells expressing intracellular IL17A, IL17F, and IL22^75^, as well as abolished production of these canonical Th17 cytokines by purified memory CD4^+^ T cells in vitro under non-polarising conditions and by naïve and memory CD4^+^ T cells cultured under Th17-polarising conditions^75, 76^ (Table 1, Figure 1).

AR HIES can also be caused by biallelic mutations in *DOCK8*, encoding Dedicator of Cytokinesis 8, a guanine nucleotide exchange factor involved in regulating the activity of Rho family GTP enzymes^77^. DOCK8 deficiency causes a combined immunodeficiency, including severe susceptibility to bacterial, viral, and fungal infections.^77^ Whilst there are many cellular defects that underpin multiple infectious diseases, DOCK8-deficiency compromises the ability of naïve CD4^+^ T cells to differentiate into Th17-type cells^78^. Interestingly, DOCK8 has been reported to associate with STAT3, and STAT3 activation is impaired in DOCK8-deficient cells^79^. These findings suggest a causal link between STAT3-deficiency, DOCK8 deficiency and CMC.

Activating *STAT1* GOF mutations cause immune dysregulation characterised predominantly by recurrent bacterial, viral and fungal infections (CMC), with the latter affecting almost all patients in the form of oropharyngeal, cutaneous, esophageal, genital, or onycho-mycoses^80^ (Table 1). CD4^+^ T cells from these patients exhibit reduced, but not abolished, Th17 cells^71, 73, 80^ (Table 1). Whilst the mechanism underlying defective Th17 generation due to *STAT1* GOF remains incompletely determined, it is possible that stronger or sustained cellular response to the STAT1-dependent cytokines IFN-γ, IFN-α and IL-27 inhibits Th17 development^80, 81^ (Figure 1). However, as impaired Th17 differentiation is also observed for naïve *STAT1* GOF CD4^+^ T cells cultured in vitro under Th17 conditions but in the absence IFN-γ, IFN-α and IL-27^73^, additional cell-intrinsic mechanisms that impair Th17 generation must exist (Figure 1). For instance, binding of STAT3 to specific target genes and induction of STAT3-target genes including *RORC*, *IL17*, *IL22*, and *SOCS* are impaired in immune cells from patients with *STAT1* GOF mutations^82^. Similarly, studies in mice revealed that STAT1 and STAT3 can counter-regulate the function of each other^83^. For instance, deletion of *Stat1* enhances STAT3 activation, while reduced IL-6-mediated activation of STAT3 augments STAT1 activation. These effects are regulated by differential induction of SOCS3^83^.

Studies of individuals with IEI due to *STAT3* DN, *RORC* LOF, *ZNF341* LOF or *STAT1* GOF variants have provided valuable insights into infectious susceptibility and a cellular and molecular explanation for their clinical features. Specifically, these findings revealed the essential intrinsic roles of STAT3, RORγt and ZNF341 in regulating differentiation of naïve CD4^+^ T cells into effector Th17 cells, and the opposing effect of hyper-active STAT1 on this pathway. The findings also provide a mechanism for extreme susceptibility to *Candida albicans* and the high incidence (~85%) of CMC in these patients (Table 1). Furthermore, determining the molecular etiology of disease due to *STAT1* GOF variants enabled targeted therapies of these individuals with JAK inhibitors. Whilst STAT1 GOF causes a panoply of clinical presentations, CMC is a prominent feature. Treatment of affected individuals with JAK inhibitors including ruxolitinib or baracitinib resulted in significant and rapid (1–8 weeks) improvement in CMC in >90% of patients. Thus, JAK inhibition as a precision medicine is highly effective for treating severe CMC in the setting of STAT1 GOF^84, 85^.

### Cytokine Receptors:

While the findings described above established critical roles for STAT3 signaling in generating and/or maintaining Th17 cells, they did not directly identify the upstream STAT3-activating cytokine(s) required for this process. However, this has now been achieved by the identification and analysis of patients with inactivating mutations in specific cytokine receptors that signal through STAT3, namely IL-12R, IL-23R, IL-6R and IL-21R.

Patients with recessive mutations in genes encoding receptors for IL12 (*IL12RB1*/*IL12RB2*) or IL-23 (*IL12RB1/IL23R*) predominantly suffer from mycobacterial disease^86–88^. However, ~30% of individuals with pathogenic variants in *IL12RB1* or *IL23R*, but not *IL12RB2*, also endure recurrent fungal infections^86–89^ (Table 1). IL-12RB1- or IL23R-deficient memory CD4^+^ T cells exhibit significantly reduced production of IL-17A, IL-17F and IL-22 ex vivo and in vitro, yet production of these cytokines by IL12RB2-deficient cells was intact^71, 87, 88^ (Table 1, Figure 1). Similar findings were obtained when in vitro differentiation of naïve CD4^+^ T cells from these patients was examined^73, 87, 88^. These findings implicate IL-23 in generating Th17 cells in humans. However, the reduction in Th17 cells in IL-12RB1/IL23R-deficiency was not as severe as in STAT3-deficiency^71, 73, 87, 88^, and the penetrance of CMC was incomplete^72, 87, 88, 90^, supporting a role for additional cytokines in Th17 generation.

Interestingly, a similar proportion of patients with IL-21R-deficient patients also have problems controlling fungal infections, although not strictly CMC (~30%)^90–93^ (Table 1). *IL21R* LOF mutations dramatically reduced, but did not abolish, the in vivo, ex vivo and in vitro generation of Th17 cells^71, 73, 90^ (Table 1). Heterozygous DN mutations in *IL6ST*, encoding the shared gp130 component of the IL-6 family of cytokines, result in a clinical condition similar to AD-HIES due to STAT3^DN^ variants, with the exception of a very low incidence of CMC (1/9 patients, Table 1)^94^. Consistent with this, naïve CD4^+^ T cells bearing *IL6ST* variants produced similar levels of IL-17A and IL-17F as naïve CD4^+^ T cells from healthy donors following culture under in vitro Th17 polarizing conditions. *IL6ST* mutant memory CD4^+^ T cells also secreted intact amounts of IL-17A, even though the proportions of these cells expressing Th17 cytokines was modestly reduced^94^ (Table 1). Taken together, it appears that IL-23 (via IL-12Rβ1/IL23R) and IL-21 are the predominant STAT3-activating cytokines involved in generating Th17 cells in humans, thereby explaining the incidence of CMC in ~30% of individuals with these pathogenic gene mutations (Figure 1). These data also highlight functional redundancy and/or co-operativity between these cytokines in CD4^+^ T cell differentiation inasmuch that the complete absence of signalling via IL-21, IL-23 or IL-6 is insufficient to completely abolish induction of Th17 cells.

### DECTIN1:

Dectin-1 (or C-type lectin domain family 7 member A, CLEC7A) is a C-type lectin receptor (CLR), mostly expressed by myeloid cells, which recognizes β-glucans present in the cell wall of certain pathogens including fungi and some bacteria^95, 96^. Upon β-glucan recognition, Dectin-1 recruits and activates the spleen tyrosine kinase SYK, engaging the Caspase recruitment domain-containing protein 9 (CARD9) protein to promote production of pro-inflammatory cytokines (IL-6, IL-23) by dendritic cells, that induce differentiation of CD4^+^ T cells into IL-17-producing T cells^95, 97^. In 2009, three adult siblings with onychomycosis caused by *Trichophyton rubrum* (dermatophytosis) and vulvovaginitis caused by *C. albicans* were reported to be homozygous for a deleterious allele of *DECTIN1* (p.Y238*)^98^. The heterozygous parents had a milder, but similar clinical phenotype. The authors showed impaired induction of IL-17-producing T cells in some experimental conditions, including upon stimulation with *C. albicans*. However, the p.Y238X *DECTIN1* allele is a common polymorphism, with a frequency of ~7% in European populations and of up to 40% in the San population of South Africa^98^ (http://hapmap.ncbi.nlm.nih.gov.proxy.insermbiblio.inist.fr/). Considering the frequency of the p.Y238* *DECTIN1* allele and that of CMC (~10^−5^), it remains difficult to consider Dectin-1 deficiency as a genetic etiology of CMC^99^.

### CARD9:

The adaptor protein CARD9 is predominantly expressed in myeloid cells and transduces signals downstream of several pattern recognition receptors, including CLRs, and is critical for antifungal immunity (Figure 1) ^100^. AR CARD9 deficiency was first described in 2009 in a large multiplex Iranian consanguineous family with CMC and possibly central nervous system (CNS) *Candida* disease^101^. Since then, >80 CARD9 deficient patients have been reported^102^. Approximately one third display superficial fungal diseases (CMC or dermatophytosis), whereas most are highly vulnerable to life-threatening invasive (e.g. affecting bones, CNS, soft tissues) fungal infections, frequently caused by *Candida* spp., dermatophytes, or black fungi, but are resistant to other microorganisms^102^ (Table 1). CARD9-deficient macrophages, dendritic cells, or peripheral blood mononuclear cells (PBMC) exhibit reduced production of cytokines (including IL-6, IL23) and chemokines when stimulated in vitro with various fungal ligands (Figure 1). CARD9-deficient neutrophils have a selective killing defect toward some but not all fungi tested^103^. Impaired CARD9-dependant chemokine production resulting in impaired neutrophil recruitment – and subsequent reduced neutrophil killing - to the sites of infection^104–106^, combined with impaired CARD9-dependant cytokine production, contribute to the susceptibility to invasive fungal diseases seen in CARD9 deficient patients^102, 103^. Variable production of IL-17, measured *ex vivo* or *in vitro* after stimulation of CARD9-deficient PBMCs, suggests CARD9 is important, but not critical, for Th17-cell differentiation, possibly through production of IL-23 by myeloid cells, accounting for the incomplete penetrance of CMC in patients with AR CARD9 deficiency^102, 103^.

## Inborn errors of the IL-17A/F response pathway

As described above, dissection of the molecular and cellular bases of IEI impairing production of IL-17A/F in patients with CMC paved the way for the identification of inborn errors of IL-17A/F-mediated immunity that impair the ability of target cells to respond to the effector cytokines produced by Th17 cells, thus conferring CMC in otherwise healthy individuals.

In 2011, a candidate gene approach identified AD IL-17F and AR IL-17RA deficiencies, each in a single family, as the first genetic causes of isolated CMC^103, 107^ (Table 1, Figure 1). A pathogenic variant in *IL17F* was identified in five relatives from an Argentinian family with early-onset CMC. The index patient also had recurrent upper respiratory tract infections, asthma, and episodes of furunculosis since infancy. A 9-month-old female relative heterozygous for the variant was also reported but was asymptomatic at the time of the study. All patients tested displayed normal proportions of IL-17A- and IL-22-producing T cells *ex vivo*. The pathogenic variant impaired binding of IL-17F to its receptor on fibroblasts. Consequently, fibroblasts or keratinocytes stimulated with mutant IL-17F homodimers or heterodimers (i.e. mutant IL-17F/wild type IL-17F, mutant IL-17F/IL-17A) showed impaired production of IL-6 and Gro-α, demonstrating the mutant IL-17F was both hypomorphic and DN on wild-type IL-17F- and IL-17A-mediated responses^107^ (Table 1). A second family of Tunisian-German descent has also been reported, with a woman and her son carrying a heterozygous *IL17F* variant and both presenting CMC from early childhood but otherwise healthy^108^.

In parallel, AR complete IL-17RA was reported in a child born to first cousin-parents of Moroccan descent. The patient suffered from early-onset CMC and cutaneous *S. aureus* infection, and carried a homozygous nonsense variant affecting the extracellular domain of IL-17RA. Additional homozygous mutations have since been reported in 27 patients with AR IL-17RA deficiency, from 14 unrelated kindreds originating from Morocco, Turkey, Japan, Saudi Arabia, Algeria, Argentina, and Sri Lanka^103, 109, 110^. All patients suffered from early-onset CMC. In addition, ~75% also presented staphylococcal skin diseases, and ~30% developed recurrent bacterial respiratory tract infections^103, 107, 109, 110^ (Table 1). AR complete IL-17RC deficiency was subsequently identified in three unrelated patients born to consanguineous families originating from Turkey and Argentina, with early-onset CMC in the absence of any other infectious phenotype, staphylococcal disease in particular^111^.

*TRAF3IP2* encodes ACT1, a key signaling adapter functioning downstream of the IL17R complex. AR ACT1 deficiency was identified in 2013 in two siblings born to consanguineous Algerian parents, with early-onset CMC and recurrent severe skin and scalp disease caused by *S. aureus*^112^ (Table 1). Both patients carried a homozygous missense mutation of *TRAF3IP2*^112^. Nine additional patients born to non-consanguineous Spanish or Portuguese parents or consanguineous Indian, Lebanese or Turkish parents, carrying biallelic variants of *TRAF3IP2* with early-onset CMC, severe skin, scalp, and pulmonary diseases were subsequently reported^113–117^. Fibroblasts of all IL-17RA-, IL-17RC-, and ACT1-deficient patients tested failed to respond to IL-17A and IL-17F homodimers and heterodimers in terms of IL-6 or Gro-α production^107, 109–112^ (Table 1). IL-17RA- and ACT1-deficient PBMCs, unlike IL-17RC-deficient PBMCs, also failed to respond to IL17E/IL-25^110, 112, 114^, which signals through IL-17RA/IL-17RB in an ACT1-dependent manner^118^.

JNK1 belongs to the MAPK signaling pathway and is involved in various signaling pathways, including the IL-17 pathway^13^. AD JNK1 deficiency was recently reported in a multiplex French family with syndromic CMC. Three patients from three generations suffered from early-onset CMC, mucocutaneous *S. aureus* infections, and a connective tissue disorder and carried a private heterozygous LOF variant of *MAPK8* encoding JNK1^119^ (Table 1). Accordingly, patients’ fibroblasts displayed impaired cellular responses to IL-17A and IL-17F. JNK1 also acts downstream of TGFβ1, which contributes to human Th17 differentiation *in vitro*^57, 120, 121^ (Table 1). Consistent with this, proportions of *ex vivo* and *in vitro*-differentiated Th17 cells were reduced, but not abolished, in the patients compared to healthy donors. AD JNK1 deficiency caused CMC by haploinsufficiency, impairing production of and cellular responses to IL-17A/F^119^ (Table 1, Figure 1).

Thus, these five IEI due to pathogenic variants that prevent production of IL-17F homodimers or IL-17A/F heterodimers, abolish expression/function of IL17RA or IL17RC (receptors for IL-17A/F) or cripple signaling downstream of IL-17RA/C further highlight the critical role of IL-17A- and IL-17F-mediated immunity in mucocutaneous protection against *Candida* and *S. aureus*. They also suggest that IL-17A- and IL-17F-dependent immunity is otherwise redundant for protection against fungi other than *Candida*, bacteria other than *S. aureus*, viruses, or against invasive candidiasis or staphylococcal disease^103, 122^.

## Autoimmune phenocopies: autoantibodies against IL-17A and IL-17F

Since the early 1980s, autoantibodies (auto-Abs) against cytokines have progressively emerged as important host factors in susceptibility to specific infectious diseases. By blocking the function of their target cytokines, these auto-Abs underlie infectious phenocopies of IEI of the corresponding cytokine or response pathway^12^. CMC is one of the three most common clinical manifestations of patients with autoimmune polyendocrine syndrome type 1 (APS-1, also called APECED syndrome), and is usually the earliest to appear^123^. In 2010, two independent studies reported that, irrespective of age, >90% of APS-1 patients tested had high serum titers of neutralizing IgG auto-Abs against at least one of the IL-17 cytokines (IL-17A, IL-17F, IL-22; Table 1, Figure 1). None of the healthy controls, healthy heterozygous relatives, or other patients with various autoimmune or endocrine syndromes tested in parallel had such auto-Abs^124, 125^. The only exception were two patients with thymoma who displayed auto-Abs against IL-17A and IL-22, and who were the only two patients with CMC of these 35 thymoma patients tested^125^. Levels of auto-Abs against IL-17 cytokines were already high before the onset of CMC^125^. Furthermore, since 2012, CMC has been described in ~2–20% of patients treated with therapeutic antibodies blocking IL-17A and/or IL-17F or IL-17RA^126^. This “relatively” low proportion likely reflects incomplete blocking of IL-17 cytokines in IL-17-competent individuals from birth. Nevertheless, this observation provides near-experimental proof that auto-Abs neutralizing IL-17 cytokines can underlie CMC, and provides strong support for a role of auto-Abs against IL-17 cytokines in the onset of CMC in APS-1 patients^126^.

## Conclusion

The identification of various inborn errors of IL-17A/F immunity or auto-Abs against IL-17A/F, as well as the use of therapeutic antibodies blocking IL-17A/F function, have altogether demonstrated the essential role of human IL-17A and IL-17F in protective immunity against mucocutaneous infections with *C. albicans*. These studies also suggested that IL-17RA-dependent immunity is involved in mucocutaneous protection against *S. aureus*, but is largely redundant in host defense against other common microbes, including other bacteria and fungi, as well as viruses. These “experiments of Nature” further demonstrate the importance of deciphering the molecular and cellular mechanisms underlying human infectious diseases, thereby shedding light on their critical and redundant function in human immunity, and illuminating possible pathways to improved therapeutic intervention for people affected by these IEI and microbial infections.

## Figures and Tables

**Table 1:** Inborn errors of immunity and phenocopies resulting in compromised IL-17-mediated immunity and fungal infections

Genetic defect	IL-17A/F signaling	IL-17A/F production;	CMC	*Staph* disease	Other infectious diseases	Auto-immunity	Refs
**AD IL-17F deficiency**	Impaired	IL-17A: normal; IL-17F: NT	+	− (+)	−	−	^107, 108^
**AR IL-17RC deficiency**	Abolished	Normal	+	− (+)	−	−	^111^
**AR IL-17RA deficiency**	Abolished	Normal to increased	+	+	− (+)^a^	−	^107, 109, 110^
**AR ACT1 deficiency**	Abolished	Normal to increased	+	+	−	− (+)^b^	^112–117^
**AD JNK1 deficiency**	Impaired	Low	+	+	+/− ^c^	−	^119^
**AR IL-12p40 deficiency**	NT (presumed normal)	Low ex vivo	+/−^d^	−	mycobacteriosis and Salmonellosis	−	^127^
**AR IL-12Rβ1 deficiency**	NT (presumed normal)	Low ex vivo and after in vitro differentiation	+/−^e^	−	mycobacteriosis and Salmonellosis	−	^71–73, 86^
**AR IL-12RB2 deficiency**	NT (presumed normal)	Normal	−	−	mycobacteriosis, pulmonary tuberculosis	−	^87^
**AR IL-23R deficiency**	NT (presumed normal)	Normal ex vivo, low after in vitro differentiation	−	−	mycobacteriosis	−	^87, 88^
**AR IL-6R deficiency**	NT (presumed normal)	subnormal ex vivo, low after in vitro differentiation	−	+	+ Bacterial diseases	−	^128, 129^
AR GP130 deficiency*	NT (presumed normal)	Low to normal ex vivo	−^f^	+	+ Bacterial diseases	−	^130, 131^
**AD GP130 deficiency**	NT (presumed normal)	Normal ex vivo, low after in vitro differentiation	−^g^	+	+ Bacterial diseases and secondary aspergillosis), ^h^	−	^94^
**AR IL-21R deficiency**	NT (presumed normal)	Impaired	− (+)^i^	−	+ Bacterial, parasitic, fungal	−	^90–93^
**AR RORγ deficiency**	NT (presumed normal)	−	+	−	+ BCGosis	−	^74^
**AD STAT3 deficiency**	NT	−	+	+	Bacterial diseases	−	^66, 68–70, 72^
**AR ZNF341 deficiency**	NT	Impaired	+	+	Bacterial disease, Pulmonary disease	−	^76^
**AD JNK1 deficiency**	Impaired	Low to normal	+	+	Bacterial diseases	−	^119^
**AR DOCK8 deficiency**	NT (presumed normal)	Impaired	++	+	Viral (Molluscum, HSV), bacterial	−	^77–79^
**AD STAT1 GOF**	NT	Low	++	++	Bacterial, viral, fungal	+	^71, 73, 80, 81^
**AR CARD9 deficiency**	NT	Impaired to normal	+	−	Invasive fungal diseases	−	^101, 102^
**Anti IL-17 AutoAb**			+			+	^12, 124, 125^

NT: not tested

a 2 cases of suspected pulmonary tuberculosis or tuberculous meningitis, respectively;

b discoid lupus erythematosus reported in one consanguineous multiplex family;

c urinary tract infections (pyelonephritis and cystitis);

d ~6% of patients with AR IL-12p40 deficiency display CMC

e about 25% of patients with AR IL-12Rβ1 deficiency display CMC;

f one patient had aphthous tongue ulcer suggestive of an undefined fungal lesion;

g one patient reported with onychomycosis;

h one patient reported with five episodes of shingles

i one patient reported with esophageal candidiasis

## Abbreviations:

DN: dominant negative
GOF: gain of function
IEI: inborn errors of immunity
LOF: loss of function
STAT: signal transducer and activator of transcription
CMC: chronic mucocutaneous candidiasis
AR: autosomal recessive
AD: autosomal dominant
JAK: Janus activating kinase
CARD9: Caspase recruitment domain-containing protein 9
